# ^17^O NMR spectroscopy of crystalline microporous materials

**DOI:** 10.1039/d1sc00552a

**Published:** 2021-02-25

**Authors:** Sharon E. Ashbrook, Zachary H. Davis, Russell E. Morris, Cameron M. Rice

**Affiliations:** School of Chemistry, EaStCHEM and Centre of Magnetic Resonance, University of St Andrews St Andrews KY16 9ST UK sema@st-andrews.ac.uk rem1@st-andrews.ac.uk

## Abstract

Microporous materials, containing pores and channels of similar dimensions to small molecules have a range of applications in catalysis, gas storage and separation and in drug delivery. Their complex structure, often containing different types and levels of positional, compositional and temporal disorder, makes structural characterisation challenging, with information on both long-range order and the local environment required to understand the structure–property relationships and improve the future design of functional materials. In principle, ^17^O NMR spectroscopy should offer an ideal tool, with oxygen atoms lining the pores of many zeolites and phosphate frameworks, playing a vital role in host–guest chemistry and reactivity, and linking the organic and inorganic components of metal–organic frameworks (MOFs). However, routine study is challenging, primarily as a result of the low natural abundance of this isotope (0.037%), exacerbated by the presence of the quadrupolar interaction that broadens the spectral lines and hinders the extraction of information. In this Perspective, we will highlight the current state-of-the-art for ^17^O NMR of microporous materials, focusing in particular on cost-effective and atom-efficient approaches to enrichment, the use of enrichment to explore chemical reactivity, the challenge of spectral interpretation and the approaches used to help this and the information that can be obtained from NMR spectra. Finally, we will turn to the remaining challenges, including further improving sensitivity, the high-throughput generation of multiple structural models for computational study and the possibility of *in situ* and *in operando* measurements, and give a personal perspective on how these required improvements can be used to help solve important problems in microporous materials chemistry.

## Abstract 2

Cost-effective and atom-efficient isotopic enrichment enables ^17^O NMR spectroscopy of microporous materials to be used to probe local structure and disorder and to explore chemical reactivity.

## Graphical abstract

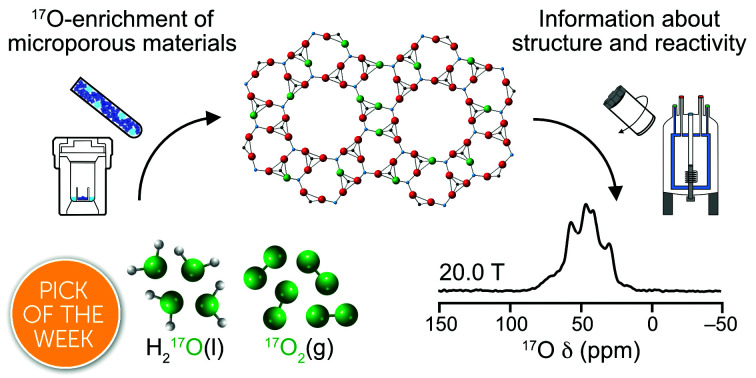

## Introduction

**Fig. 1:**
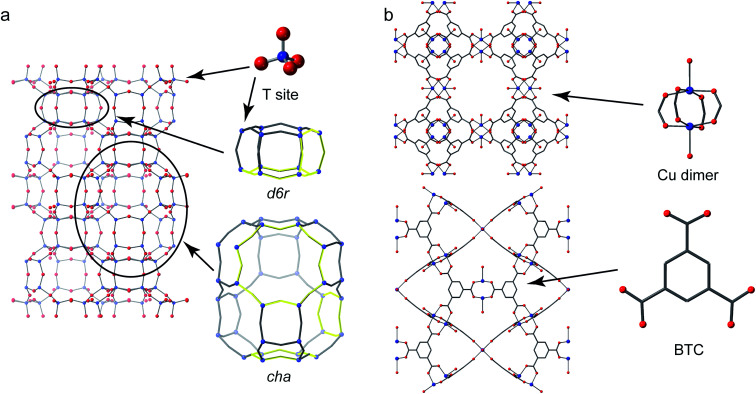
(a) The structure of CHA zeolite showing the *d6r* and *cha* cage secondary building units (SBUs) with four-, six- and eight-membered rings highlighted. (b) The structure of the Cu-based MOF HKUST-1 (viewed down the (010) and (110) directions) showing the Cu “paddlewheel” dimer and benzene tricarboxylate (BTC) linker building units.

Crystalline microporous materials are defined as solids that have ordered structures and form frameworks containing channels, cavities and pores that are of similar scale to small molecules (*i.e.*, less than 2 nm in diameter).^1^ The class of microporous solids is dominated by two main families of materials: zeolites and metal–organic frameworks (MOFs), shown in Fig. 1.

Zeolites are generally silicate- or phosphate-based solids (the latter sometimes referred to as zeotypes) that contain tetrahedral units, which are fully connected through oxygen atoms to form open frameworks.^1^ Partial substitution of, for example, silicon by aluminium in aluminosilicate zeolites imparts a negative charge to the framework that needs to be balanced by cationic species (often called extraframework cations) that can be found in the pores and channels of the solid. It is the combination of the chemistry of the framework and extraframework cations with the microporous architectures that leads to some exceptional properties that are utilised in many different industrial applications, from heterogeneous catalysis to ion exchange.^2^

Metal–organic frameworks have completely different chemistry to zeolites but share many of the topological features of the porosity.^3^ MOFs usually comprise a metal ion or metal cluster that acts as a node and a polytopic organic ligand that links the nodes together, resulting in a porous open framework. Probably the most common organic linkers are those based on carboxylates but almost any linker that has the possibility to form coordinate bonds to two or more metals can be used: common ones include imidazolates and pyrazolates.

For any porous material, the nature of the internal surface of the pores is vitally important as it controls the interaction with any guest species that are adsorbed. The amount of accessible internal surface area is often measured using gas adsorption experiments. For MOFs the quoted surface areas can be as high a several thousand square metres per gram of material, which has led to great interest in these materials for high-capacity gas adsorption.^4,5^ For comparison, in zeolites this figure is usually several hundred square metres per gram. The chemistry of the internal surface is also important and techniques that can probe the local environments around the atoms that form the surfaces can provide pivotal information that aids in our understanding of the structure and function of the materials.

In general, the detailed structural characterization of microporous materials poses an interesting, but considerable, challenge. The presence of compositional, positional and temporal disorder^6^ in, for example, the framework cations, any structure directing agents (SDAs) used in the synthesis, charge-balancing cations and anions, water or absorbed guest molecules, means that the use of a single characterization technique is unlikely to yield a complete picture. In this regard, nuclear magnetic resonance (NMR) spectroscopy, with its sensitivity to the local chemical environment, and to motion on timescales over several orders of magnitude, has been widely used alongside more conventional diffraction-based approaches.^7,8^ In many cases, the information on long-range order, often readily obtained from diffraction, can be linked to the atomic-scale detail provided by NMR spectroscopy through the use of first-principles calculations (an approach often referred to as NMR crystallography).^9,10^

Although ^1^H, ^13^C, ^29^Si, ^27^Al and ^31^P NMR have been widely employed for studying microporous solids,^11–15^ there is growing interest in the potential application of oxygen NMR experiments. In particular, for zeolites, oxygen atoms predominantly line the surface of the internal pores that play such a vital role in reactivity, while oxygen is also a common component of many MOF materials – often joining the metal nodes to the organic linkers, and potentially providing information on both components of these hybrid materials. Oxygen is also a key component of some SDAs, of charge-balancing hydroxyl groups, of Brønsted acid sites, in water and is present in many guest molecules.

The twin challenges of improving sensitivity and resolution are often said to be continual themes in solid-state NMR spectroscopy, and this is perhaps never truer than for ^17^O (the only NMR-active isotope of oxygen).^16^ The high spin quantum number (*I* = 5/2) leads to additional spectral broadening from the quadrupolar interaction that cannot be removed by conventional sample rotation (known as magic-angle spinning).^17^ Perhaps a more significant challenge is the extremely low natural abundance of ^17^O, which is only 0.037%, severely limiting sensitivity and making it difficult, if not impossible, to study all but the simplest systems. This problem, however, is perhaps also the greatest opportunity of ^17^O NMR; the isotopic enrichment that can be used to improve sensitivity also offers an ideal prospect for exploring the stability and chemical reactivity of frameworks and for gaining insight into the mechanism of their formation.

In this Perspective, we outline the current state-of-the-art in ^17^O NMR spectroscopy of microporous solids. We discuss the different methods used for cost-efficient isotopic enrichment, the challenges of acquiring and interpreting high-resolution spectra, how these can be addressed using cutting-edge experimental approaches and illustrate the information on structure and reactivity that can be obtained with examples for some key systems. Finally, we look to the future – considering the greatest challenges that remain, the recent developments that may help tackle these, and when, and indeed if, the true potential of ^17^O NMR spectroscopy for microporous materials is likely to be realized.

## Approaches for efficient ^17^O enrichment

**Fig. 2:**
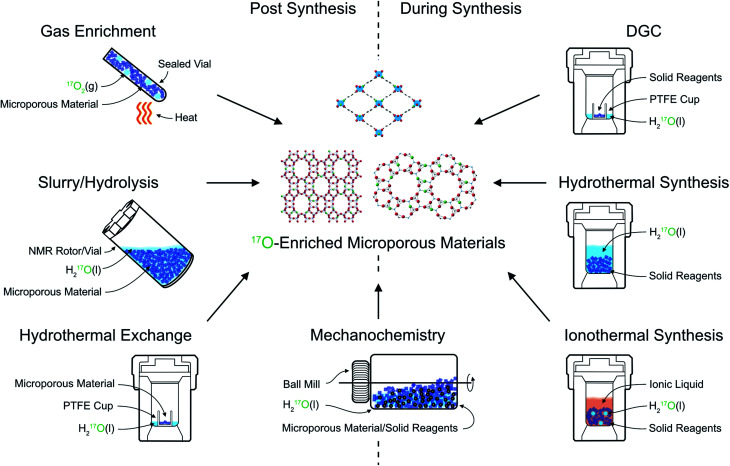
Schematic showing approaches for ^17^O enrichment of microporous materials during and post synthesis.

The primary approach used to overcome the inherently poor sensitivity of natural abundance ^17^O NMR spectroscopy is isotopic enrichment. However, the high cost of isotopically enriched reagents (*e.g.*, 90% H_2_^17^O(l) @£2000 per mL or 70% ^17^O_2_(g) @£3000 per L) often requires new synthetic procedures (or the adaption of existing ones), to ensure that enrichment is both cost effective (reduces the absolute amount of ^17^O used) and atom efficient (ensures as much as possible of this is then incorporated into the final product). For example, many microporous solids are prepared using hydrothermal approaches,^18^ with water present in significant excess, incurring significant cost and considerable waste of enriched reagents. The methods commonly employed for enrichment can be divided into two distinct types: post-synthetic enrichment of an existing microporous material and enrichment that takes place during synthesis (or as part of a chemical reaction), as shown schematically in Fig. 2. In many cases, the desire is for uniform enrichment (to allow quantitative measurements to be made) and experimental conditions must be carefully optimised to achieve this. In contrast, if enrichment is more selective, with very different rates or absolute levels of enrichment for different species, insight into chemical reactivity and mechanism can be obtained.

The earliest ^17^O enrichment of microporous materials (LTA and FAU zeolites), by Oldfield and co-workers in 1986,^19^ exploited two different approaches: (i) a “hydrothermal” exchange process and (ii) through the (albeit inefficient – see later) use of H_2_^17^O(l) in a traditional zeolite synthesis. For zeolites, the hydrothermal exchange process involves combining a pre-prepared framework material with H_2_^17^O(l) in an autoclave (or similarly sealed vessel), and heating at autogenous pressure at temperatures typically between 125 and 220 °C. Some authors report enrichment in just 3 hours (for LTA, LSX and SOD),^20,21^ while others use much longer durations (4–6 weeks for LTA, FAU and STI).^22–24^ The amount of H_2_^17^O(l) added in each case also varies – typically between 50% (ref. 19) and 100% (ref. 22 and 24) of the mass of zeolite to be enriched. While the conditions chosen may well affect the absolute level of enrichment, this is very challenging to measure in the final product (see later), and it is not always stated in early work. It is also not always clear how/if the levels seen would vary with reaction time for any particular material (and therefore why, and indeed how, the conditions were chosen). Note that the three letter codes are used to designate zeolite topology (LTA, FAU *etc.*, above) and give no information about chemical composition, and so even materials with the same topology might have significantly different enrichment chemistry. Hydrothermal exchange processes have been shown to enrich both Si–O–Si and Si–O–Al framework linkages efficiently (although the absolute uniformity of enrichment is not considered in most studies), making it a generally useful approach for ^17^O NMR studies of zeolite frameworks.

Post-synthetic ^17^O enrichment using a hydrothermal exchange process has also been shown for MOFs. For Sc-MIL-53, direct synthesis of an ^17^O-enriched material using a dry gel conversion (DGC) approach (chosen to limit the amount of solvent required – see later) proved challenging, resulting in the preferential (and sometimes exclusive) formation of an alternative, small-pore MOF, Sc_2_(BDC)_3_.^25^ Enrichment was therefore carried out through hydrothermal exchange of Sc-MIL-53 pre-prepared using a conventional hydrothermal approach (which is not itself easily amenable for direct enrichment owing to the cost of the high volume of solvent required). Sc-MIL-53 was placed in a PTFE cup inside an autoclave that contained 130 μL of H_2_^17^O(l) and was heated to 200 °C under autogenous pressure for 72 h. This provided enrichment of both hydroxyl and carboxyl O species, without breakdown of the framework, and an estimated ^17^O enrichment level of ∼25% in the final product.^25^ It should be noted that the poorer thermal and hydrolytic stability of many MOFs (compared to most zeolites) requires careful consideration of exchange conditions, balancing the level and rate of enrichment against the potential for framework degradation.

Perhaps the most common method for ^17^O enrichment of zeolites is high-temperature, post-synthetic exchange with ^17^O_2_(g): an approach that is also widely applied to many other inorganic oxides.^16^ For zeolites, this was first reported for Na-ZSM-5 by Oldfield and co-workers in 1989.^26^ For such reactions, a dehydrated (and calcined) zeolite is heated in an atmosphere of ^17^O_2_(g) at high temperature (usually >500 °C) for typically between 5 and 24 hours.^26–34^ An elevated temperature (usually close to the point at which the framework becomes unstable and begins to degrade) is chosen to ensure enrichment is as rapid and as uniform as possible although, where relevant, conditions under which dealumination occurs must be avoided. It is generally assumed that this approach provides uniform levels of ^17^O throughout the material (*i.e.*, equal levels of enrichment for Si–O–Si and Si–O–Al linkages^27,35^), although it should be noted that recent work on oxides showed that for some materials much higher temperatures (∼900 °C) were required for this to be the case, and it may be that this is not possible to achieve for some porous solids without risking significant framework breakdown.^36^ It is, perhaps, reasonable to suggest that enrichment using this approach is likely to be more uniform than that achieved using some of the other approaches described. Both siliceous^33^ and aluminosilicate zeolites have been enriched using post-synthetic exchange with ^17^O_2_(g). For aluminosilicate zeolites, the framework can be in its protonated (acid) form,^27,35^ or metal-exchanged (*e.g.*, with Na^+^,^27,28^ K^+^,^31^ Cs^+^,^34^ Ca^2+^,^31,32^ or Sr^2+^,^34^*etc.*) form, providing a very flexible approach. Typical enrichment levels are between 10 and 20%, depending on the exact conditions and the isotopic composition of the ^17^O_2_(g) used (usually between 40 and 70% ^17^O_2_). However, the O_2_-rich atmosphere and high temperatures employed make this method unsuitable for enrichment of zeolites and phosphate frameworks in their as-made forms (*i.e.*, with the organic SDA present) or for MOFs, and alternative approaches are required.

In some zeolites, it is not necessary to use hydrothermal conditions to obtain enrichment. In recent work, it was demonstrated that a number of zeolites frameworks (MOR, FER and CHA) could be isotopically enriched in ^17^O simply by exposure to H_2_^17^O(l) under ambient conditions, revealing the very surprising reversible lability of all framework bonds, even at room temperature.^27,37^ These reactions were also carried out *in situ* inside the NMR rotor (mixing ∼ 50 mg of zeolite with 50–100 μL of 90% H_2_^17^O(l) in approach sometimes referred to as slurrying), enabling the rate of enrichment to be followed as a function of time for different species (see later). Although enrichment was surprisingly rapid (*i.e.*, within a day) much longer durations (>30 days) were required to obtain a more uniform distribution of ^17^O.^27,37^ Although not published, we have seen in recent work that a similar slurrying approach enables enrichment of Si–O–Al linkages in SAPOs, but that enrichment of the SAPO Al–O–P bonds (or indeed of AlPO frameworks) is considerably slower at room temperature.^38^

It is also possible to exploit the inherent susceptibility of some zeolite materials towards hydrolysis to isotopically enrich in ^17^O. Bignami *et al.*^39^ used H_2_^17^O(l) in the acid hydrolysis of germanosilicate UTL zeolite to produce a second, isotopically enriched, but different zeolitic product. These reactions were carried out *in situ*, *i.e.*, in a sealed polymer insert within a 4 mm ZrO_2_ rotor, to enable insight into the mechanism of the ADOR (Assembly, Disassembly, Organisation, Reassembly) process^40–42^ (see later for more detail) to be obtained.^39,43^

Many microporous solids, including zeolites, phosphates and MOFs, are typically prepared using hydrothermal synthesis,^18^ in which frameworks are crystallised at high temperatures (>100 °C) in a PTFE-lined autoclave at autogenous pressure, typically for >12 hours in the presence of water. While this should be an effective method to ensure random incorporation of ^17^O into a material, the volume of water required is often many mL, leading to high overall costs and low overall enrichment efficiency. It would be possible to use this approach if the reaction volume could be reduced significantly, but while this can be accomplished for some materials, in some cases this can lead to different reactivity and the formation of different products. Alternatively, costs could be decreased by reducing the level of enrichment in the water used. In early work by Oldfield, enrichment of zeolites and phosphates was carried out using this traditional approach.^19,44^ For Na–Y and Na–A zeolites, 2–3 g of 50% enriched H_2_^17^O(l) was used, leading to 0.8–1.2 g of product that was 22–32% enriched in ^17^O (assuming equal distribution of ^17^O between all reagents).^19^ Notably, for Na–X a much larger volume of water was used (6.8 g) but with a reduced enrichment level of 20% for the synthesis alumino- and gallosilicate materials.^19^ For phosphate frameworks (AlPO-5, AlPO-11 and AlPO-17), between 1 and 2 g of 50% H_2_^17^O(l) was employed, and the enrichment level was retained after calcination and removal of the SDA (at 500 °C for 3 h).^44^ Although clearly a possible route to (hopefully uniform) enrichment, the high costs and low atom efficiency suggest direct hydrothermal synthesis is not the optimum route for routine ^17^O studies.

One approach to remove the need for a significant excess of water synthesis is ionothermal synthesis, where an ionic liquid is used as both the solvent and as the SDA.^45,46^ This has primarily been employed in the formation of phosphates,^46,47^ with more limited examples in the literature of the synthesis of silicon-based zeolites^48,49^ and MOFs.^50^ The first ionothermal synthesis of phosphate frameworks (SIZ-1, SIZ-3, SIZ-4 and SIZ-5) was reported in 2004, using 1-methyl-3-ethyl imidazolium bromide as the ionic liquid.^47^ This procedure is similar to hydrothermal synthesis, with starting materials combined with the ionic liquid and heated (typically at temperatures of ∼200 °C) at autogenous pressure. It was shown by Griffin *et al.* that the inclusion of small (μL) quantities of H_2_^17^O produced isotopically enriched products.^51^ Using 50 μL of 35% enriched H_2_^17^O(l) for 3 g of aluminium isopropoxide (and 1-methyl-3-ethyl imidazolium chloride as the ionic liquid), gave a SIZ-4 product 4% enriched in ^17^O. This enrichment level was retained upon calcination and removal of the SDA. The lack of excess water in this approach ensures that it is atom efficient and, as with hydrothermal synthesis, enrichment is presumed to be uniform. Although the level of enrichment shown by Griffin *et al.*^51^ is low, the increase of a factor of 100 over natural abundance levels reduces the experimental time by a factor of ∼10 000, enabling conventional and two-dimensional NMR spectra to be acquired on a reasonable timescale. It was noted that increasing the enrichment level of the H_2_^17^O(l) to 90% would further increase the enrichment level by a factor of 2.6 (an additional time saving of a factor of 6.7 in experimental time) whilst retaining reasonable cost.^51^ Although ionothermal synthesis has, so far, only been used for ^17^O enrichment of phosphate frameworks it could, in principle, be used for any material that can be prepared in this way. Care must be taken, however, as the amount of water that can be added to the reaction without changing the reaction pathway and/or products is often restricted. In theory, one could imagine also extending this idea to solvothermal synthesis (an approach that is applicable to MOFs in particular), potentially widening the range of materials that could be enriched.

Other approaches that reduce the amount of water required in the synthesis of microporous materials are clearly potential candidates for efficient ^17^O enrichment. First suggested in 1990, DGC was proposed to reduce the solvent required and eliminate waste liquids in the synthesis of zeolites (specifically ZSM-5) by separating the amorphous gel/solid phase from the liquid phase.^52^ This method has been extended to the synthesis of other microporous materials including phosphates, such as SAPO-18, SAPO-34 and AlPO-11, and MOFs, such as Al-MIL-53 and HKUST-1.^53–57^ As DGC uses the minimum amount of solvent required for a successful synthesis, this makes it a potentially useful and cost-effective approach for producing ^17^O-enriched frameworks, with H_2_^16^O(l) simply replaced by H_2_^17^O(l) in the reaction. The small volumes of solvent used mean that high levels of H_2_^17^O(l) enrichment (50–90%) are typically required. Practically, DGC is carried out in a PTFE-lined autoclave, which contains 50–200 μL of H_2_^17^O(l). The solid reagents are mixed in an additional PTFE cup which is then placed inside the autoclave so that the reagents are not in direct contact with the liquid water, before the autoclave is sealed and heated under autogenous pressure at temperatures usually between 150–200 °C.^52–55^ This approach often leads to uniform enrichment (as for example, seen for MIL-53,^25,54^ where carboxyl and hydroxyl O species are both efficiently enriched). However, for AlPO-11, preferential enrichment of O bound to two of the three unique P sites in the final product was observed.^55^ This was explained, on the basis of ^17^O NMR, by the formation of a layered AlPO intermediate in which O species were exchanged with ^17^O in the water vapour. Access to some O species in this phase is restricted due to hydrogen bonding with the SDA, and subsequent conversion to AlPO-11 led to absence of ^17^O in the coordination sphere of P1.

A potentially interesting route for ^17^O enrichment of framework materials is mechanochemistry.^58^ This approach, which has been used for the synthesis of both organic and inorganic materials, is known for its advantages over traditional synthetic routes, including its more environmentally friendly nature, faster reaction times, lower temperatures and reduced solvent requirements. It is possible that mechanochemical approaches could be used to introduce ^17^O to starting materials or to enrich frameworks during synthesis or post synthetically. For example, many MOFs contain linkers which bind to the metal nodes through a carboxylate functional group, as seen in MIL-53, CPO-27 and UiO-66.^59^ Recent work by Laurencin and co-workers^60^ showed how a variety of organic molecules, including the terephthalic and trimesic acid linkers used in MOFs could be isotopically enriched by ball milling. This involved activation of the carboxylate by reaction with 1,1′-carbonyl-diimidazole, followed by hydrolysis with 1.5 equiv. of 41% H_2_^17^O(l) when milling for 5 min (at a rate of 25 Hz). Enrichment levels of between 3 and 11% were obtained, allowing conventional ^17^O NMR spectra to be acquired in a few hours and multi-dimensional NMR spectra to be acquired overnight. This was shown to be an extremely efficient process, with 70 μL of 41% H_2_^17^O(l) used to enrich 60 mg of four different acids (at a cost of ∼€50). In order to then synthesise an enriched MOF framework, care must be taken to design a subsequent synthetic approach that does not result in any significant loss of the incorporated isotope.

In recent work by Rainer *et al.*,^61^ mechanochemistry was applied in zeolite hydrolysis, with 100 μL of 40% H_2_^17^O(l) added during ball milling (at 150 rpm) of 500 mg of (unenriched) Ge-UTL in an ADOR reaction,^39,43^ leading to an overall enrichment level in the daughter zeolite of 10%. In this context, mechanochemistry offers a simple and cost-efficient route to enrichment of novel zeolitic products that are difficult to synthesise and enrich using more traditional routes. Direct synthesis of microporous materials with limited solvent or so-called “solvent free” reactions (where no solvent is intentionally, and additionally, included but water is often present on the surfaces or within the precursors) is also possible using mechanochemical methods.^62–64^ Although to our knowledge this approach has not yet been used in the context of ^17^O enrichment of porous solids, solvent free synthesis may offer a simple and efficient route to framework enrichment using pre-enriched starting materials (such as the enriched carboxylic acids described above), while mechanochemical reactions with reduced solvent would be a cost-effective route to direct enrichment if a small volume of highly enriched H_2_^17^O(l) was used.

The theoretical maximum level of enrichment of a microporous material can be determined by considering the proportions of ^16^O and ^17^O present in all starting materials and reagents, and assuming a uniform distribution of the two between all chemical species in all products and by products. However, it can be challenging to measure the absolute level of enrichment obtained using NMR spectroscopy, with the extremely low natural abundance of ^17^O precluding a direct quantitative comparison with NMR spectra of unenriched materials for all but the simplest of oxides. A more accurate determination of the enrichment level can be achieved using mass spectrometry, as employed for MIL-53 by Bignami *et al.*,^25^ where enriched samples embedded in indium mounts and covered with a 30 nm coating of gold were exposed to a ^133^Cs focussed primary ion beam. Secondary ions were extracted (at 10 kV) for ^16^O, ^17^O and ^18^O and results obtained with a standard error of ∼1% for enriched materials. Although this approach is too expensive to be employed routinely, it is possible to compare the sensitivity of ^17^O NMR spectra for systems where enrichment levels have been accurately determined to those of other materials to estimate the level of enrichment obtained.

## Experimental measurement and spectral interpretation

NMR spectra of solids are typically broadened by the orientation dependence, or anisotropy, of the interactions that affect the nuclear spins.^7^ In solution, these are averaged to an isotropic value by rapid molecular tumbling, leading to spectra with inherently high resolution and easy separation of chemically different species. In the solid state, this problem is usually tackled by physical rotation of the sample around an axis inclined at an angle *χ* = 54.736° to the external magnetic field, in an approach termed magic-angle spinning (MAS).^7,65^ For sufficiently rapid rotation (*i.e.*, at frequencies larger than the magnitude of the spectral broadening the interaction causes), the anisotropic components of the shielding, dipolar coupling and *J* coupling interactions can be effectively removed, resulting in high-resolution, isotropic NMR spectra. As the size of the sample holder, or rotor, decreases, faster MAS rates are available; however, the potential increase in resolution this provides has to be balanced against the loss in sensitivity from the smaller sample volume, and so the fastest available MAS rate is not always employed.

**Fig. 3:**
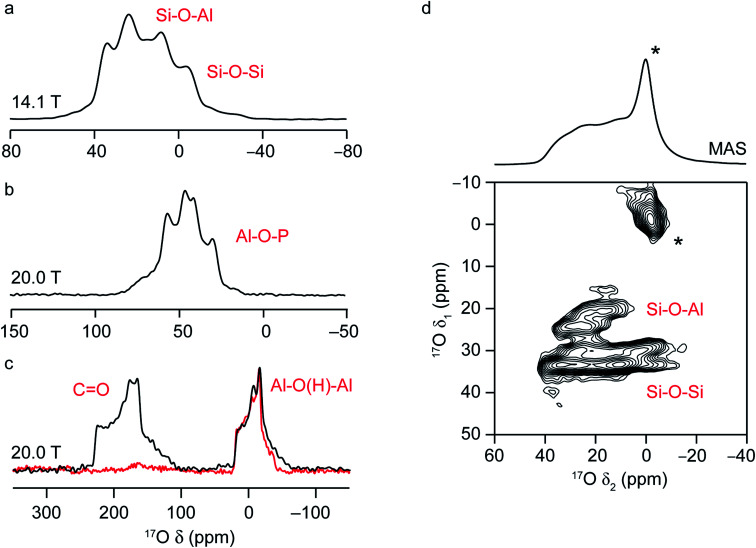
^17^O MAS NMR spectra of (a) aluminosilicate CHA zeolite, (b) calcined CHA-type aluminophosphate framework, SIZ-4 and (c) calcined Al-MIL-53. The external field strengths at which spectra were recorded are shown in the figure. In (c), the ^1^H–^17^O CP MAS NMR spectrum is shown in red. (d) ^17^O (14.1 T) MAS and MQMAS of a hydrated CHA zeolite (after slurrying with H_2_^17^O(l)).

Solid-state NMR spectra for nuclei with high spin quantum number, such as ^17^O (*I* = 5/2), are additionally broadened by the quadrupolar interaction.^16,17^ This interaction between the nuclear electric quadrupole moment and the electric field gradient at the nucleus is often significant, leading to anisotropic broadening over many hundreds of kHz or even MHz. The tensor that describes the interaction can be parameterised by its magnitude, *C*_Q_ (the quadrupolar coupling constant) and its asymmetry or shape, *η*_Q_, which lies between 0, for an axially symmetric tensor, and 1. In many cases, the magnitude of the quadrupolar interaction is such that its effect on the Zeeman nuclear spin energy levels has to be treated using second-order perturbation theory, leading to a more complex angular dependence of the anisotropy, and field-dependent anisotropic broadening that cannot be completely removed by MAS. This results in limited resolution, with spectra often containing overlapped powder-pattern lineshapes, as shown in Fig. 3. However, the dependence of the MAS NMR spectrum not just on the isotropic chemical shift, *δ*_iso_, but also on the quadrupolar parameters, *C*_Q_ and *η*_Q_, provides additional opportunities to probe the local structure and to differentiate spectral signals. This can also be seen in Fig. 3a–c, where the ^17^O MAS NMR spectra of a chabazite (CHA) aluminosilcate zeolite, the calcined CHA-type aluminophosphate framework SIZ-4, and a calcined MOF, Al-MIL-53, are shown. The two chemically different O species in the zeolite framework (Si–O–Al and Si–O–Si) have different isotropic chemical shifts (18–20 ppm and 28–30 ppm, respectively) but also different quadrupolar couplings (∼4 and ∼5.3 MHz).^37^ The four Al–O–P species in SIZ-4 have much larger *C*_Q_ (5.8–6.0 MHz) and shifts between 50 and 64 ppm.^51^ For Al-MIL-53, there is a significant shift difference between the carboxyl signal at ∼203 ppm, and that for the bridging hydroxyl oxygens (∼20 ppm), and also a difference in quadrupolar couplings (∼7 MHz and ∼5.5 MHz).^25,66^

The presence of the quadrupolar interaction can also pose challenges for the acquisition of quantitative spectra, which are necessary to determine the uniformity of enrichment and to extract accurate information on the proportion of different species present.^7,17^ In addition to accounting for relaxation (as is required in all quantitative NMR experiments), it is also necessary to take into account the different nutation rates (*i.e.*, the different responses to the application of a radiofrequency pulse) of species with different *C*_Q_.^36^ This is usually achieved by choosing a pulse with very short duration (or small “flip angle”), reducing sensitivity but ensuring a quantitative spectrum can be obtained. Note that in cases where species with large and very small *C*_Q_ are present simultaneously, it might also be necessary to account for the contribution of the satellite transitions to the MAS spectrum, as described in ref. 36 and 67.

Although the quadrupolar interaction can provide structural information, it is often desirable to remove this anisotropic broadening completely and record a high-resolution spectrum. In early work, this was achieved using the composite sample rotation techniques, dynamic angle spinning (DAS)^68^ and double rotation (DOR),^69^ where the sample is rotated around two angles (sequentially for DAS and simultaneously for DOR). Although more widely employed for ^17^O NMR of simple oxides, ceramics and minerals, these approaches have been applied to zeolites and phosphates.^20,25,33,51,70–73^ However, DAS and DOR are technically challenging to implement, requiring specialist and expensive hardware. The most popular approach for the acquisition of high-resolution spectra for quadrupolar nuclei is multiple-quantum (MQ) MAS,^74^ a two-dimensional experiment that correlates a multiple-quantum (usually triple-quantum, *m*_*I*_ = +3/2 ↔ *m*_*I*_ = −3/2) transition with the observable (*m*_*I*_ = +1/2 ↔ *m*_*I*_ = −1/2) transition. The result of this experiment (after appropriate processing) is a two-dimensional spectrum that contains a series of ridge-like lineshapes, one for each distinct species, with an isotropic spectrum free from quadrupolar broadening obtained from a projection onto the *δ*_1_ axis. Analytical fitting of the quadrupolar-broadened lineshapes obtained from cross-sections parallel to *δ*_2_ for each ridge allow values of the NMR parameters (*δ*_iso_, *C*_Q_ and *η*_Q_) to be extracted independently for each signal. As an example, the ^17^O MQMAS NMR spectrum of a hydrated CHA zeolite (enriched after slurrying with H_2_^17^O(l)) is shown in Fig. 3d and contains four distinct framework signals; two Si–O–Al and two Si–O–Si,^37^ and a significant increase in resolution from the MAS spectrum (also shown). The larger quadrupolar coupling of the Si–O–Si sites is clear from the broader lineshapes for these signals in *δ*_2_.

MQMAS is commonly applied to acquire high-resolution spectra of quadrupolar nuclei in many materials, owing to the ease of its implementation and the need for only conventional MAS hardware. Its biggest drawback, however, is its inherently poor sensitivity – typically between 3 and 10% of a simple MAS spectrum, leading to lengthy spectral acquisition. As an example, the ^17^O MAS NMR spectrum of CHA in Fig. 3d was acquired in ∼2 hours, whereas the MQMAS spectrum took ∼20 hours to obtain. Furthermore, the efficiency of MQMAS is strongly dependent on the *C*_Q_ value (as well as on the strength of the radiofrequency pulses and the MAS rate) leading to non-uniform excitation of different signals.^75,76^ Over the years, there have been a number of attempts to improve the sensitivity of this approach, primarily using composite pulses,^77–80^ to improve the efficiency of the multiple-quantum conversion. Although sensitivity improvements of factors of 2–3 are often obtained (as seen in ^17^O NMR of LTA and FAU zeolites^23^), most of these approaches are sample dependent and can be time consuming to implement and optimise (more so when sensitivity is low). The satellite-transition (ST) MAS experiment^81,82^ offers an alternative approach to high-resolution spectra from quadrupolar nuclei. Related conceptually to MQMAS, this method exploits different single-quantum (*m*_*I*_ = ±3/2 ↔ *m*_*I*_ = ±1/2) transitions within the spin system, resulting in inherently higher sensitivity than MQMAS (often by a factor of 4 to 8). However, STMAS is technically more challenging to implement, requiring an extremely accurate setting of the spinning angle and precise timings of the pulses. Interestingly, however, the satellite transitions, and therefore STMAS spectra, are sensitive to motion on the microsecond timescale and, hence, are a potentially useful probe of dynamics.^83^ This has been demonstrated for ^27^Al and ^71^Ga NMR of zeolites^84–86^ and ^17^O NMR of minerals^87^ – and so could be a potentially useful tool for studying motion of *e.g.*, guest molecules in future ^17^O NMR studies of microporous solids.

For many ordered inorganic materials, MQMAS enables resolution of crystallographically distinct sites – a good example of this can be found in ref. 88, where the ^17^O MQMAS spectrum of a silicate mineral resolves the signals from the six distinct O sites, despite their similarities. However, if sites are so similar that their NMR parameters are almost identical, complete resolution is not always possible. This is the case for the ^17^O MQMAS spectrum of calcined SIZ-4 (the corresponding MAS NMR spectrum for which is shown in Fig. 3b), where two signals are seen in the isotropic spectrum at 20.0 T for the 4 distinct O species (corresponding to O1/O4 and O2/O3 in the CHA framework, respectively).^51^ For more disordered materials, where a range of NMR parameters may be present for a specific site, depending on the variation in the local environment, the resolution of crystallographically distinct species can be challenging. In these cases, however, resolution of chemically different species is usually possible. For example, in the ^17^O MQMAS spectrum of zeolite CHA in Fig. 3d,^37^ signals from Si–O–Al and Si–O–Si species are very clearly resolved. Although two different Si–O–Si signals are also resolved, there are four crystallographically distinct sites, so not all signals are fully resolved.

Once spectra can be acquired with sufficient resolution and sensitivity, there still remains the challenge of understanding and interpreting the complex lineshapes that are encountered, and of assigning the signals to specific chemical environments. While the existence of large databases of NMR parameters is common for liquid-state ^1^H and ^13^C NMR spectroscopy, such information is not as readily available for inorganic solids, and particularly for less commonly studied nuclei such as ^17^O. In some cases, insight can be obtained using empirical correlations, where NMR parameters for one or more model systems are correlated with structural parameters (usually from prior diffraction studies) before the structure–parameter relationships derived are used to predict parameters or to assign unknown signals. See the next section for examples of this approach. However, it is often the case that these relationships work well for systems that are very similar to the model system but are poorly transferrable to a wide range of materials. Further insight can be gained using more advanced experimental approaches. In particular, experiments that transfer magnetisation between nuclei can indicate spatial proximity or through-bond connectivity of spins (for transfers *via* the dipolar coupling and *J* coupling, respectively).^7^ As an example, the ^17^O NMR spectrum of Al-MIL-53 (ref. 25) acquired using cross polarisation (where magnetisation is transferred from ^1^H to ^17^O *via* the dipolar coupling) in Fig. 3c (red) shows the selective excitation of the signal from the hydroxyl O. These magnetisation transfer experiments can be extended to two dimensions, enabling site-specific information on the species that are linked by the relevant interaction to be obtained. When both spins involved in the transfer are quadrupolar, the spin dynamics are considerably more complex and quantitative measurements are hard to achieve, although qualitative information on spatial proximity can still be obtained.^17^ A number of different ^1^H–^17^O experiments (*e.g.*, HETCOR, REDOR and TRAPDOR) have been used to probe Brønsted acidity in zeolites, in work pioneered by Grey and co-workers.^28,29,89^ Huang and co-workers used ^17^O/^27^Al and ^17^O/^31^P REDOR experiments to aid assignment of the O signals seen during the formation of an AlPO-11 aluminophosphate framework,^55^ and to probe the cation distribution in SAPO-34.^56^

In recent years, there has been growing interest in the use of calculations to predict NMR parameters alongside experiment to aid assignment and interpretation of spectral lineshapes.^9,10^ In most cases, these are carried out using density functional theory (DFT), owing to the balance of efficiency and accuracy that it provides, with many studies performed using periodic planewave codes^90^ to exploit the inherent translational symmetry of crystalline solids. For microporous materials, it is important to accurately account for dispersion forces in the calculations, and semi-empirical dispersion correction schemes are often added to the commonly-used PBE functional.^10,91^ In the absence of these, particularly flexible materials can adopt different and often much more open conformations than expected (as demonstrated in the literature for phosphate frameworks and for Sc-MIL-53 (ref. 10)), and host–guest interactions are poorly represented, leading to incorrect prediction of NMR parameters. For disordered solids sets of structural models with different atomic positions or different compositions can be easily and efficiently compared to experiment.^8,10^ While relatively little computational work has focused exclusively on ^17^O NMR of microporous materials, there are many studies demonstrating the accuracy of predicted ^17^O NMR parameters in ceramics, minerals, biomaterials *etc.*^9^ Computation of ^17^O NMR parameters has been used successfully to assign signals in mixed-metal MOFs, phosphate frameworks and zeolites.^25,51,54,56,66,92^ There is, however, considerably more scope for closer interaction between ^17^O NMR experiments and computation with recent improvements in hardware and software, and with the growing expertise within the scientific community.

## Extracting information on structure and disorder using ^17^O NMR

The sensitivity of NMR to the atomic-scale environment ensures that chemically different O species (*i.e.*, Si–O–Si, Si–O–Al, Al–O–P, Si–O(H)–Al, *etc.*) within a framework solid have different NMR parameters, enabling signals to be distinguished within the spectrum, and the type and relative proportions of each species present to be determined. Changes in the number of species present can be monitored as the composition varies, guest molecules are loaded or chemical reactions take place, providing information on structure and reactivity that can, ultimately, be linked to physical and chemical properties. For zeolites and zeotypes, there has been a significant focus on understanding how (and indeed if) the NMR interactions are correlated to specific geometrical parameters, with the aim that such relationships (if present) can then be used to determine detailed structural information directly from an NMR spectrum. However, there are challenges with this approach; it can be difficult to extract accurate NMR parameters in the presence of significant second-order quadrupolar broadening and the distribution of local environments present in disordered materials. This can be eased both through the study of more ordered systems (*e.g.*, zeolites with Si/Al = 1, high silica materials or calcined AlPOs),^33,44^ and the use of high-resolution experiments, such as DAS, DOR and MQMAS. Given these challenges, many authors have also exploited computation, where the effect of varying a single structural parameter (often using a simplified structural model consisting of a small cluster of atoms) can be more easily studied.^93,94^ While this allows a deeper fundamental understanding of the origin of the NMR parameters and their dependence on geometry, it can be difficult to relate this back to the more complex, disordered (and often hydrated) frameworks studied in experiment.

For T–O–T′ linkages in zeolites and zeotypes (where T/T′ represent the atom(s) on neighbouring tetrahedral framework sites) the nature of the coordinated cations, the O–T distances and the T–O–T′ bond angle should have the most significant effect on ^17^O NMR parameters. Early work by Oldfield^44^ (on both silicate minerals and zeolites) established a dependence of the ^17^O *C*_Q_ on the electronegativity of the bonded T-site cation (and, therefore, on the average ionicity of the O–T bonds). Correlations between the ^17^O quadrupolar parameters (*C*_Q_ and *η*_Q_) and the Si–O–Si bond angle were demonstrated computationally in a series of publications by Grandinetti and co-workers,^93–95^ and were shown to hold for experimental NMR parameters extracted for the ten ^17^O sites in high-silica ferrierite (clarifying earlier work by Bull *et al.*^33^). It was also shown, however, that slightly different correlations were observed for different types of silica materials, as a result of the dependence of the ^17^O *C*_Q_ also on the Si–O distance. This results in NMR being a very sensitive probe of the detailed local environment, but one that could become challenging to interpret as the complexity of the system increases. Experimental studies of Na–A and (Na,K)-LSX zeolites found correlations between the ^17^O *δ*_iso_ and both the Si–O–T angle and hybridisation (*i.e.*, the s character of the O hybrid orbitals).^21,72,96^ However, care must be taken when attempting to generalise these relationships, as other work has shown the exact isotropic shift observed is dependent on the framework topology and also on the proximity of water and the nature of the charge balancing cation present.^32,70,96^ In comparison to zeolites, there have been fewer studies of the dependence of ^17^O NMR parameters on local geometry for phosphates, owing, in part, to the complexity of as-made materials (which contain different, and often disordered, structure directing agents, charge-balancing anions and water) and the challenges and costs of enrichment. Oldfield^44^ predicted ^17^O *C*_Q_ values for Al–O–P linkages based on known correlations with the average ionicity of the two bonds and the difference in ionicity between the two. More generally, it was shown that more ionic linkages exhibit smaller *C*_Q_, while as the difference in ionicity of the two bonds increases the orientation of the quadrupolar tensor rotated toward the more covalent bond.

**Fig. 4:**
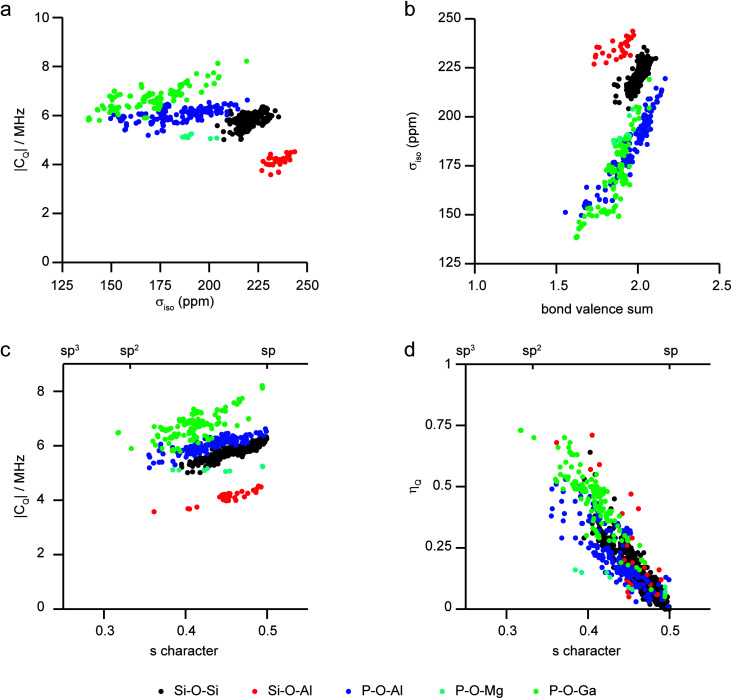
Plots showing calculated ^17^O NMR parameters for a range of model framework solids,^86,91,97–99^ coloured by T–O–T′ linkage. (a) |*C*_Q_| against *σ*_iso_, (b) *σ*_iso_ against bond valence sum, (c) |*C*_Q_| and (d) *η*_Q_ against the s character of the O bonding.

As the level of disorder in a system increases, the challenge of resolving distinct sites and extracting accurate NMR parameters grows. Furthermore, the average structural picture that is produced by diffraction-based methods also means that structural parameters cannot easily be extracted for individual coordination environments in disordered solids. One solution is to turn to computation, where site-specific NMR parameters can be easily linked to the site-specific geometry for a range of substituted frameworks. This approach (using periodic planewave DFT) has been successfully applied to consider the dependence of ^29^Si and ^31^P NMR parameters on the local geometry in zeolites and phosphates, respectively.^97–99^Fig. 4 shows a summary of calculated ^17^O NMR parameters for T–O–T′ linkages in a range of silicate/aluminosilicate zeolites and substituted phosphate frameworks from our previous work^86,91,97–99^ (with charge-balancing H added where required). Fig. 4a plots ^17^O |*C*_Q_| against *σ*_iso_, and shows that chemical variation of the linkage leads to differences in both of these NMR parameters with, for example, Ga–^17^O–P oxygens having higher *C*_Q_ and Si–^17^O–Al oxygens having the highest shielding (*i.e.*, lowest chemical shifts). For *σ*_iso_, a poor general correlation is seen with the T–O–T angle (not shown), but for each O type there is a good correlation with the bond valence sum (*i.e.*, a measure of the ionic/covalent nature of the bonding at O) with increased shielding (Fig. 4b). As shown in Fig. 4c, there is a correlation between the ^17^O |*C*_Q_| and the s character of the O–T bonds (defined as cos *θ*_T–O–T′_/(1 − cos *θ*_T–O–T′_))^95^ for each type of linkage (although the exact *C*_Q_ exhibited depends on the nature of the atoms bonded). There is a clear (and general) correlation of *η*_Q_ with s character of the O–T bonds, with *η*_Q_ approaching 0 (*i.e.*, axial symmetry) as the s character approaches 0.5 (or as *θ* approaches 180°), as shown in Fig. 4d. Although the NMR interactions are correlated with the local geometry, these relationships are clearly complicated, and unambiguous prediction of a structural model is not possible from these alone.

In addition to bridging T–O–T′ linkages in the framework, zeolites may also contain defects such as silanols or aluminols and/or non-bridging oxygens. The low levels in most frameworks makes these species more difficult to identify or characterise, and little ^17^O NMR work has been attempted on these so far in conventional frameworks. However, in ADOR reactions, a much larger number of non-bridging oxygens are found in the hydrolysed intermediate phases produced. Work by Bignami *et al.*^39^ confirmed the presence of Si–OH species with ^17^O *δ*_iso_ = ∼23 ppm and *C*_Q_ = ∼3.4 MHz, although it should be noted high-power decoupling was required to observe this signal at all in the MQMAS experiments. Low temperature (108 K) ^1^H–^17^O CP experiments, where dynamics are restricted, showed a significant increase in signal, suggesting such species might generally be difficult to observe if present only as defects in conventional frameworks using ^17^O NMR at room temperature.

The NMR parameters for any non-bridging oxygens present in zeolites are likely to vary with any extra-framework cation present. Although not yet studied in depth for zeolites, work from Oldfield and co-workers on alkaline Earth silicate minerals^100^ showed a dependence of the ^17^O *δ*_iso_ for non-bridging oxygens on the cation radius and of the *C*_Q_ on the cation electronegativity (with *C*_Q_ increasing as the electronegativity increased). Although the variation in *C*_Q_ with cation electronegativity was similar to that seen for bridging oxygens in the same materials, the absolute values of *C*_Q_ were lower (by ∼2 MHz). In zeolites, the NMR parameters observed will depend also on the level of hydration, and it seems likely more extensive investigation will be required to understand these relationships in more detail.

Much of the work on zeolites described above was carried out on non-protonated or ion-exchanged materials. However, the ability to study the Brønsted acid sites that are important in catalysis is crucial, but this has been more challenging, likely as a result of a larger ^17^O *C*_Q_ and the mobility of H (and hence rapid relaxation) in hydrated zeolites. In 2005, Grey and co-workers used high-field double resonance experiments (and reference to previous computational work^101^) to unambiguously identify the Si–O(H)–Al signal in dehydrated zeolite HY (Si/Al = 2.6), an important cracking catalyst.^30^^1^H–^17^O CP spectra (with short contact time) enabled Si–O(H)–Al oxygens to be selectively observed, giving a lineshape that could be fitted using *C*_Q_ = 6.6 MHz, *η*_Q_ = 0.8 and *δ*_iso_ = 28 ppm. ^1^H–^17^O REDOR and TRAPDOR experiments showed dephasing consistent with a large dipolar coupling, proving the H was directly attached to the framework O in the dehydrated zeolite. The acidity of this site was demonstrated through the change in ^17^O NMR parameters upon exposure to acetone-d_6_.^30^ Weak Si–O–(H)–Al signals were later observed in ^17^O MQMAS spectra of dehydrated HY,^35^ but two-dimensional ^1^H–^17^O HETCOR experiments were able to resolve two different Si–O(H)–Al signals,^89^ corresponding to the Brønsted acid sites in the supercages and sodalite cages (which had differences in ^1^H and ^17^O *δ*_iso_ of 0.7 and 3 ppm, respectively). Similar Si–O(H)–Al signals were also identified in high silica HZSM-5 (Si/Al = 25), although weaker REDOR dephasing confirmed longer O–H bonds and/or increased H mobility was present in this material.^89^ Lower temperature ^1^H–^17^O REDOR experiments on HY and HZSM-5 were able to freeze out some of the motion that averages NMR interactions, enabling accurate O–H bond distances of 0.97–0.98 Å to be determined.^28,102^^1^H–^17^O double resonance experiments were also used to probe the accessibility of Brønsted acid sites in a MOR zeolite, using the adsorption of a trimethylphosphine-d_9_ (TMP) probe molecule.^29^^1^H–^17^O CP and HETCOR spectra showed two types of acidic hydroxyls, with longer and shorter O–H bonds, giving lower and higher values of ^17^O *η*_Q_. The loading experiments provided information on the distribution of the acid sites, showing 20–25% were located in the 8-ring channels (and therefore inaccessible to TMP), 25–30% at the intersection of the side pockets and 12-ring channels were partially accessible (*i.e.*, only at low loading), while the fully accessible remainder (45−50%) were located in the 12-ring channels.

The degree of ordering of heteroatoms in the zeolite framework (and hence the distribution of acid sites) is thought to be one of the most important factors in determining the physical and chemical properties of these materials, and the development of approaches to characterise the heteroatom distribution continues to be the focus of considerable research effort. While most investigations have focussed on ^29^Si and ^27^Al NMR, studies using ^17^O have recently started to appear in the literature. One of the most comprehensive, by Cadars *et al.*, studied the Ga/Si distribution in a natrolite-type zeolite (Rb-PST-1).^103^^17^O MQMAS experiments resolved a number of different T–O–T′ linkages, and DFT calculations were used to aid their assignment. A new computational approach^104^ was used to generate a large number (8 × 10^8^) of structural models with different possible cation arrangements within a 1 × 1 × 2 supercell, from which 8000 were randomly selected. These were ranked using a simple calculation of the electrostatic energy, with 32 low energy structures then selected for more accurate DFT calculations. ^17^O MQMAS spectra simulated using the calculated parameters showed that the signal position was dependent both on the chemical nature of the bonded cations (*i.e.*, whether Si–O–Si, Si–O–Ga or Ga–O–Ga), but also on the type of crystallographic O species present (of which there are three in Rb-PST-1). However, these relationships resulted in the overlap of signals from different species in the MQMAS spectrum, hindering unambiguous deconvolution. Notably, the work disproved that the simple assignment of all signals at low *δ*_1_ to Ga–O–Ga (as suggested in previous work on glasses),^105^ although the presence of these linkages (which contravene Lowenstein's rule) could not be ruled out completely. The computational work also enabled a detailed investigation into the dependence of the ^17^O NMR parameters on local structure, revealing a correlation between ^17^O *δ*_iso_ and the T–O–T′ angle and (unexpectedly) with the average O–T–O angle, while exact values are also affected by the distance to the nearest charge-balancing Rb cation.

Huang *et al.*, used ^17^O–^27^Al and ^17^O–^31^P REDOR and TRAPDOR experiments to assign the ^17^O NMR signals seen in AlPO-11 (AEL) and SAPO-34 (CHA).^55,56^ In both cases, NMR was used to characterise both the final product and the intermediate phases (where OAl_3_, OAl_4_, H_2_O, P–O–H and Al–O–Al species are also present) in the DGC synthesis. Significant differences in *C*_Q_ were seen for chemically different species. For SAPO-34, *C*_Q_ values of 3.5–3.6 MHz and 6.1–6.3 MHz were found for Al–O–Si and Al–O–P linkages, respectively, and the relative intensity of these signals when resolved in an MQMAS spectrum confirmed the level of Si substitution (∼25%). Much smaller *C*_Q_ values (of 1.2 and 4.2 MHz) were found for OAl_4_ and OAl_3_ in the intermediate phases, while Al–O–Al and P–O–H linkages exhibited *C*_Q_ values of 4.0 and 4.7 MHz. For SAPO-34,^56^ it was shown that the initial gel is amorphous, transforming during the first hour of heating to a fairly ordered layered AlPO phase, where only Al–O–Al (but notably not Al–O–P or P–O–H) linkages are enriched in ^17^O. A semicrystalline SAPO intermediate is formed after 4 hours, with P–O–H and Al–O–P linkages also now enriched, with the final SAPO-34 product formed after 2 days.

The O species present in MOFs are chemically more varied, resulting in chemical shifts of 0–150 ppm and 200–300 ppm for hydroxyl and carboxylate groups, respectively.^25,54,106^ The exact shift seen depends on the topology of the framework, the organic linker and the type(s) of metal present, with hydroxyls that bridge between adjacent metal cations showing the more significant variation with the latter. For example, for MIL-53 where hydroxyls bridge adjacent metals in chain, increases in *δ*_iso_ of 11 ppm and a further 113 ppm are seen on moving from Al- to Ga- and to Sc-based material. A third common oxygen species present in MOFs is the oxo group, found in metal–oxygen clusters, such as Zn_4_O in MOF-5 and Zr_6_O_4_(OH)_4_ in UiO-66.^54,106^ These can have very different shifts depending on the binding mode (*e.g.*, μ_2_-, μ_3_-, μ_4_-, *etc.*) and the type and number of coordinated metals. As an example, the oxo groups in MOF-5 (μ_4_–O^2−^ bonded to Zn) and Zr-UiO-66 (μ_3_–O^2−^ bonded to Zr) have shifts of −50 and 386 ppm, respectively.^54,106^ In some MOFs, O is also found in the linker itself, *e.g.*, the Ar–O^−^ species in Mg-CPO-27 has a shift of 87 ppm.^54^ The ^17^O *C*_Q_ is also diagnostic of the bonding environment, often being large for both carboxyls (7–8 MHz) and Ar–O^−^ (9–10 MHz), smaller for hydroxyls (4–6 MHz) and much smaller (1–2 MHz) for oxo species.^25,54,66,106^ Although the significant variation in ^17^O chemical shifts between different types of chemical environment in MOFs means that resonances are often reasonably well separated even when *C*_Q_ is large and significant second-order broadening is present, it can be considerably more difficult to resolve signals from chemically similar but crystallographically distinct species even using MQMAS. Recent work by Martins *et al.* showed how very high magnetic field (35.2 T) was needed to separate the signals from the different carboxylate oxygens in ^17^O MQMAS spectra of α-Mg_3_(HCOO)_6_ and Al-MIL-53, with experiments at 21.1 T unable to do this. Notably the 12 distinct species in α-Mg_3_(HCOO)_6_ could be resolved and were assigned using DFT calculations.^107,108^^17^O NMR spectra of a Cu(ii)-based MOF, HKUST-1 (prepared using DGC) have also been acquired. This MOF contains a paddlewheel dimer, where two Cu centres are bridged by four carboxylate groups, as shown in Fig. 1b. Although antiferromagnetic coupling in this dimer results in a *S* = 0 ground state, the presence of a low-lying excited triplet (*S* = 1) state gives rise to considerable paramagnetic shifts for the nearby ^13^C nuclei (which lie between −100 and 1000 ppm).^109^ As the O is directly bonded to the Cu atoms, the paramagnetic interaction is more significant, and wideline spectroscopy was used to acquire the anisotropically broadened lineshape, with *δ*_iso_ = 3530 ppm, and an anisotropy (span) of 3100 ppm.^57^

**Fig. 5:**
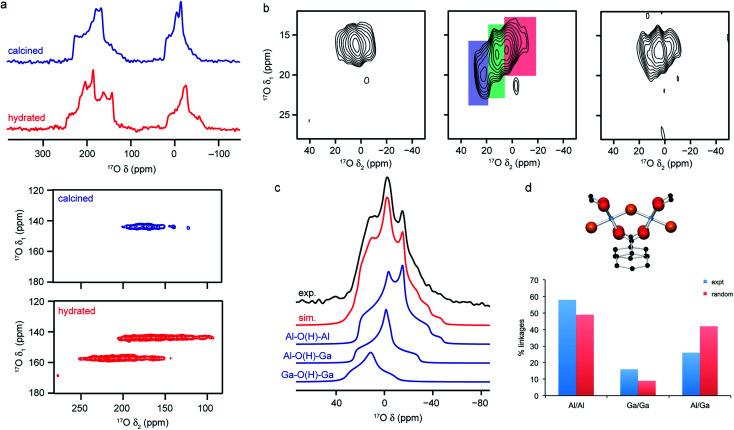
(a) ^17^O (14.1 T) MAS and MQMAS (carboxyl region only) NMR spectra of Al-MIL-53 in calcined (open pore) and hydrated (closed pore) forms. (b) ^17^O (20.0 T) MAS NMR spectra of calcined 50 : 50 (Al,Ga)-MIL-53 (hydroxyl region). (c) ^17^O (14.1 T) MAS NMR spectrum of calcined 50 : 50 (Al,Ga)-MIL-53 showing the overall fit and decomposition into components resulting from different bridging hydroxyls. (d) Schematic of the X–O(H)–X bridging hydroxyl linkage and plot comparing the proportion of different linkages extracted from (c) to those predicted assuming a random cation distribution. Figure adapted from ref. 25 and 66 with permission.

The large range of shifts seen can make it difficult to predict where ^17^O signals will appear, or to assign signals that are observed. This can be addressed using a number of approaches, including selective enrichment of different components of the MOF,^106^ comparison between chemically or structurally similar sets of materials,^25,54^ use of CP to selectively excite O bonded to H^25,54^ and prediction of NMR parameters using first-principles calculations.^25,54,66^ Although the latter is extremely powerful, care must be taken to account for any molecules adsorbed within the pores and, for flexible MOFs, any structural changes associated with these or with temperature, if accurate parameters are to be obtained. The sensitivity of the ^17^O NMR spectra to some of these changes is shown in Fig. 5a, where spectra of MAS and MQMAS spectra of calcined (open pore) and hydrated (closed pore) forms of Al-MIL-53, a breathing MOF, are shown. Calculations reveal that the two separate signals seen for the carboxylate O in MQMAS spectra of the (closed pore) hydrated form result from those involved in hydrogen bonding with guest water molecules, *δ*_iso_ = 225 ppm, and those that are not with *δ*_iso_ = 255 ppm. It is also important to account for dispersion forces in calculations (often a problem when using GGA functionals such as PBE) as these play an important role in host–guest chemistry and the structural forms adopted for flexible materials such as MIL-53.^10,66,110^ For example, for Al-MIL-53, the inclusion of dispersion forces (as a semi-empirical dispersion correction) results in the narrow pore form of the calcined MOF being predicted to be more energetically stable than the open pore form, whereas the reverse is predicted if these interactions are not accounted for.^66^

The sensitivity of the ^17^O NMR parameters to changes in the local environment can also be exploited in the study of disorder in mixed-metal materials. Of the relatively few ^17^O NMR studies of MOFs that have been published, many focus on materials containing just one metal cation, but recent work by Bignami *et al.*^25^ exploited ^17^O NMR spectroscopy to probe cation disorder in (Al,Ga)-MIL-53 materials enriched (to 11–20%) using DGC reactions. As shown in Fig. 5, ^17^O MAS and high-field (20.0 T) MQMAS spectra showed the presence of Al–O(H)–Al, Al–O(H)–Ga and Ga–O(H)–Ga linkages in a material synthesised with Al : Ga of 50 : 50, providing evidence for atomic-level mixing of metals in the framework. The relative proportions of each (extracted from a quantitative MAS spectrum, as shown in Fig. 5), showed that the composition of the final product did not reflect the composition of the starting materials, but proved there was preferential incorporation of Al (with Al : Ga of 70 : 30). The spectra also demonstrate a slight preference for clustering of like cations (with more Al–O(H)–Al and Ga–O(H)–Ga and fewer Al–O(H)–Ga than would be expected if there were a random cation distribution, as shown in Fig. 5). Bignami *et al.*^25^ noticed that the mixed-metal MIL-53 materials exhibited different breathing behaviour to the two end members, an observation investigated further by Rice *et al.*^66^ MOFs with high Al content (>70%) adopted an open pore form upon calcination (as evidenced by the presence of only one carboxylate signal in the ^17^O MQMAS spectrum), but an increase in the Ga content resulted in a mixture of the open pore and narrow pore forms (resulting in three carboxylate oxygens – one from the open pore form and two from the narrow pore form). However, MOFs with highest Ga content (>90%) adopted only the open pore form. Interestingly, the structures formed upon calcination are different from those seen for calcined, hydrated and subsequently dehydrated MOFs. All dehydrated mixed-metal materials exhibit both open pore and narrow pore forms with the proportion of the narrow pore form increasing systematically with the Ga content. Rice *et al.*^66^ also confirmed that the clustering of like cations and higher incorporation of Al over Ga seen previously does not result from the small-scale DGC synthesis, as similar effects are also observed for a (nominally) 50 : 50 (Al,Ga)-MIL-53 framework synthesised using a traditional hydrothermal approach before being enriched post-synthetically by reaction with H_2_^17^O vapour at 150 °C.^66^

## Exploring chemical reactivity using ^17^O NMR

The advantageous physical and chemical properties of many microporous materials (*i.e.*, their stability and tolerance to a range of operating conditions, their ease of use at bulk scale, their facile preparation and handling, and the tunability of their surface area, porosity and pore architecture) have led to applications in petrochemical refining, gas storage and as drug delivery agents.^1^ Microporous materials are rarely consumed in reactions; instead their role is usually to facilitate these as catalysts, ion-exchange materials or shape-separation membranes. Their high molecular weight, bulk and low framework polarity also means that they are poorly soluble and are most appropriately used in heterogenous reactions, either neat or impregnated into a substrate. Understanding how a microporous framework interacts with different disordered, and often mobile, guests is challenging, but NMR is ideally suited to provide this atomic-level detail and investigate how it changes with time. In addition, for ^17^O, the use of enriched materials during synthesis, as reagents in a subsequent reaction or simply as a solvent is able to provide additional information on the formation of frameworks (and how this might ultimately be controlled), their chemical reactivity and their stability or lability under different conditions. It is possible to follow these processes by monitoring from where enriched material is lost or gained and on what sort of timescale this occurs. This does, however, often come with caveats on the scale of reaction or volumes of solvents that can be used to ensure the process remains cost effective.

For zeolites, the level and distribution of Brønsted acid sites is key in determining reactivity, and this is often studied using probe molecules and their interaction with the reactive framework sites. As described above, Grey and co-workers used the changes in ^17^O NMR spectra upon exposure to acetone-d_6_ to study the Si–O(H)–Al sites in zeolite HY.^30^ The change in the ^17^O *C*_Q_ (from 6.6 to 5.0 MHz) was in agreement with the correlation of this parameter with O–H⋯O distance established in previous computational work on aluminosilicate clusters.^101^ When combined with information from the ^1^H–^17^O TRAPDOR experiments, an increase in the O–H Brønsted acid bond length from 0.97 to 1.02 Å could be demonstrated. The basicity-based reactivity of zeolite frameworks was studied by Freude and co-workers,^70^ who used ^17^O DOR experiments to investigate the reaction of different cation-exchanged forms of a low silica zeolite LSX with pyrrole and formic acid. Although changes in the ^17^O spectra were seen upon adsorption (and for pyrrole appeared to suggest this affected one of the four O species more than the others), it was not possible to define an unambiguous relationship between the NMR parameters and structural changes on binding, owing to the difficulties of spectral deconvolution for these disordered materials. This highlights the ongoing need to continue to develop high magnetic fields and more sophisticated multiple-resonance experiments to exploit the detailed information contained within the ^17^O NMR spectra.

**Fig. 6:**
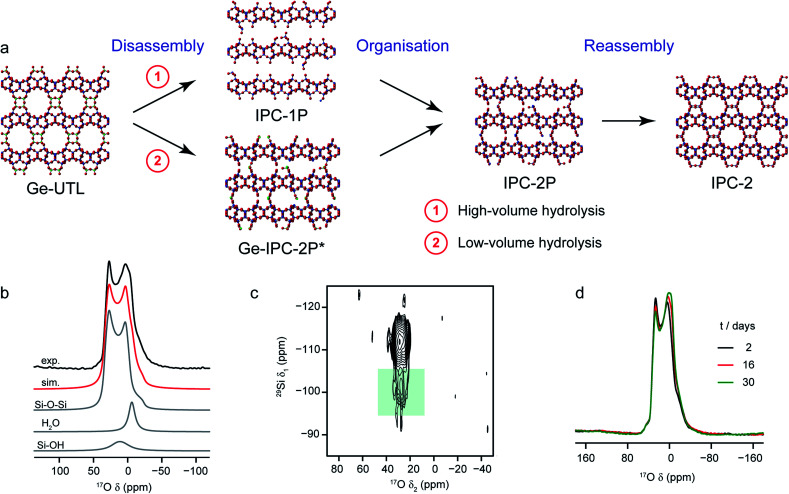
(a) Schematic showing the ADOR process for the formation of new zeolites. (b) ^17^O (14.1) MAS NMR spectrum of Ge-UTL hydrolysed with H_2_^17^O(l) for 16 days. Also shown are the lineshape fitting (red) and individual lineshapes (grey) for Si–O–Si, water and Si–OH. (c) ^17^O–^29^Si (20.0 T) D-HMQC spectrum of Ge-UTL hydrolysed with H_2_^17^O(l) for 16 hours, with the green box highlighting the correlation between Q^3^ Si–OH species and Si–O–Si oxygens only at longer recoupling times, confirming enrichment of the zeolitic layers. (d) ^17^O (14.1 T) MAS NMR spectrum of Ge-UTL hydrolysed with H_2_^17^O(l) for 16 h, acquired after 2, 16 and 30 days. Figure adapted from ref. 39, with permission.

A particularly intriguing approach is to exploit the ^17^O enrichment process itself to explore the chemical reactivity of zeolites. Maupin *et al.*,^111^ used temperature-programmed isotope exchange (with ^17^O_2_(g) in a recycling reactor at ∼550 °C), and subsequent ^17^O NMR spectroscopy to illustrate the enhanced reactivity of the NaX zeolite framework upon simple mixing with CeO_2_. The promotion of the oxygen exchange into the zeolite framework was attributed to the ability of CeO_2_ to activate O_2_ from the gas phase as superoxide species, and indicative of the presence of many small crystals of CeO_2_ on the zeolite surface. It was suggested that this process could aid the regeneration (de-coking) of the zeolite during de-NO_*x*_ conversion. The reactivity of zeolites with H_2_O (both in liquid or gaseous form) has long been of interest, as it has important implications for the structural integrity of these systems in a variety of industrial processes.^112^ In recent work using ^17^O NMR spectroscopy^39,40^ the synthesis of new zeolites *via* the ADOR^40,41^ process was investigated. Here, the structural weakness in Ge-containing zeolites is exploited by reaction with H_2_O(l), leading to selective hydrolysis and disassembly of the parent (UTL) zeolite to give intermediate layered silicate phases that are then reassembled into novel zeolites, as shown in Fig. 6a. Bignami *et al.*^39^ found that performing this reaction on a smaller absolute scale (*i.e.*, similar to the volume of an NMR rotor), and thereby reducing also the relative volume of water used, a different ADOR mechanism was observed. Although the anticipated final product (IPC-2P) was successfully formed, the reaction proceeded *via* a highly disordered intermediate (termed IPC-2P*) without any evidence for the IPC-1P phase that was well known from previous work at larger reaction volumes.^40–42^ However, as shown in Fig. 6, high-field ^17^O MAS and MQMAS NMR spectra of Ge-UTL hydrolysed with H_2_^17^O(l) revealed that hydrolytic disassembly was more extensive than previously thought. The unexpectedly high ratio of Si–^17^O–Si : Si–^17^OH species revealed that, in addition to irreversible hydrolysis of the unstable Ge–O–Si/Ge linkages, framework Si–O–Si linkages are susceptible to a reversible bond cleavage, leading to enrichment of the zeolitic layers themselves (an observation that was also supported using ^29^Si–^17^O two-dimensional double resonance experiments, where correlations between Q^3^ Si species in the interlayer space and Si–O–Si in the zeolitic layers could be seen at longer recoupling times). Furthermore, changes to the ^17^O MAS lineshape over a period of ∼30 days (Fig. 6d) after the initial reaction indicated that rearrangement of the local structure (resulting from the presence of residual water in the interlayer space) continued even after the “final” IPC-2P product was formed, despite no obvious changes in the powder XRD pattern (or indeed the ^29^Si NMR spectrum) over this period. A similar reaction was studied by Rainer *et al.*,^61^ using mechanochemistry. ^17^O NMR spectra showed enrichment of the Si–^17^O–Si linkages in UTL after only ∼30 min (at 150 rpm), with the presence of a signal thought to be attributable to Si–^17^O–Ge at short reaction times (which has not yet been observed in hydrothermal reactions). NMR spectra reveal that the mechanism of mechanochemical disassembly was different from that seen under hydrothermal conditions, with no evidence for the formation of the IPC-1P intermediate, but the formation of an (enriched) IPC-4 product alongside a notable amount of Ge^17^O_2_, most likely as a result of the very different solvent availability.

**Fig. 7:**
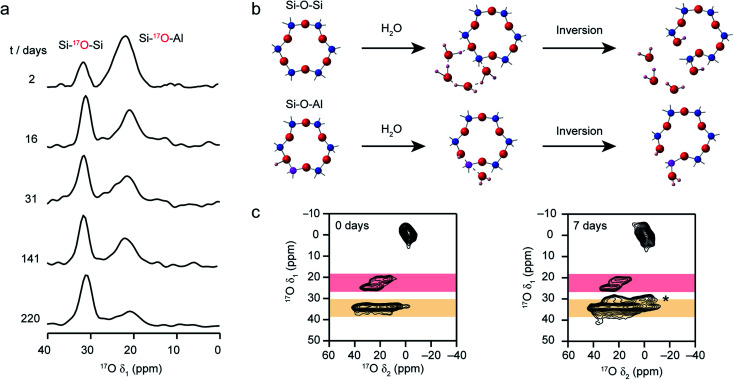
(a) Isotropic projections of ^17^O (14.1 T) MQMAS NMR spectra of H-MOR after slurrying with H_2_^17^O(l) for different times, showing the selective enrichment of Si–O–Al linkages at shorter times. (b) Schematic showing the proposed mechanism predicted using computation that leads to low energy barriers for initial bond breakage in a CHA zeolite. (c) ^17^O (14.1 T) MQMAS spectra of CHA zeolite after slurrying for 0 and 7 days, showing the growth of an additional Si–O–Si signal (marked *) at longer times. Figure adapted from ref. 27 and 37, with permission.

Recent work has also investigated the lability of conventional (*i.e.*, non ADOR-able) zeolite frameworks in ambient aqueous conditions using ^17^O NMR spectroscopy, with MAS and MQMAS spectra used to follow the (*in situ*) enrichment of MOR, FER and CHA frameworks by reaction with H_2_^17^O(l) at room temperature.^27,37^ This slurrying resulted (as described briefly above) in rapid enrichment of the zeolite framework, with evidence for Si–^17^O–Si and Si–^17^O–Al signals after just a few hours of reaction. No framework degradation was observed confirming rapid, but fully reversible, bond cleavage. The exact rate of enrichment depended on the framework topology and aluminium content, but in all cases preferential enrichment of Si–^17^O–Al linkages was observed at shorter reaction durations, as demonstrated by the ^17^O MQMAS spectra of MOR in Fig. 7a. Reactions for CHA (SSZ-13) appeared particularly rapid, an observation that was supported by *ab initio* molecular dynamics calculations.^37^ These predicted a low energy barrier for the initial bond cleavage for Si–O–Al (20–30 kJ mol^−1^) if the Brønsted acid proton is attached to the framework, but that a low barrier (∼60 kJ mol^−1^) could also be seen for three of the four crystallographic Si–O–Si oxygens in the presence of a hydrogen bonded chain of water molecules along which a proton was shuttled in a Grotthuss-type mechanism (see Fig. 7b). This mechanism was not applicable for cleavage of bonds to O2, owing to the increased steric hinderance at this site (requiring water inside the *d6r*). Interestingly, differential rates of enrichment were seen experimentally for the two Si–O–Si signals resolved in an MQMAS experiment (Fig. 7c), although these must clearly result from the overlap of signals from the four distinct O species. Although the Brønsted acid proton clearly plays an important role in bond lability, it should be noted that reversible enrichment of both Si–O–Si and Si–O–Al linkages was also seen for Na-MOR (albeit with some evidence for slower reaction rates),^27^ suggesting different mechanisms may be active depending on the system and cations present. It would appear, therefore, that not only can this slurrying approach potentially provide a cost-effective and low energy approach for enrichment of a range of microporous materials but that the rate and selectivity of enrichment could offer unique insight into chemical reactivity in future studies.

Although relatively less work has explored the chemical reactivity of phosphates using ^17^O NMR, the work by Huang and co-workers investigating the dry gel syntheses of AlPO-11 (ref. 55) and SAPO-34 (ref. 56) described above offers a tantalising glimpse into the quality and detail of the information available. The NMR spectra, and in particular, the selectivity of the enrichment, provided unique insight into the synthesis mechanism and the intermediate phases formed during these reactions. Recent work by Sun *et al.*^113^ demonstrated the reversible lability of the Al–O–P and Si–O–Al bonds in the SAPO-34 framework on exposure to steam (at 300 °C for 2 h) using ^17^O NMR spectroscopy. They were also able to exploit this dynamic behaviour to incorporate triphenylphosphine and pyridine guest molecules (both of which have kinetic diameters that are larger than the pore window), with reversible opening and closing of the bonds in the 8MR window (in a “ship in a bottle” approach). This behaviour was observed at temperatures between 100 and 300 °C, in contrast to the room temperature lability seen previously for aluminosilicates,^27,37^ with an exponential increase in exchange efficiency at higher temperatures. The authors propose a mechanism that involves only a single water molecule (unlike the hydrogen bonded chain that appears to be required for room temperature enrichment of Si–O–Si linkages in CHA above), suggesting interesting differences between the chemistry of these two isomorphous frameworks, the use of temperature and the role played by liquid water (rather than steam) in the hydrolysis reactions. There is still much to learn from ^17^O enrichment of zeolites and zeotypes, and the implications these results have for understanding the role played by these materials in catalysis and ion exchange.

NMR spectroscopy has been used to follow the synthesis of MOFs from secondary building units (SBUs),^14,114^ although to date no studies have exploited ^17^O solid-state NMR spectroscopy – probably as a result of the large solvent volume used in a conventional hydrothermal synthesis. Liquid-state ^17^O NMR spectroscopy, where the quadrupolar interaction is removed from the spectrum, has been employed to study the SBUs themselves and MOF formation. Frot *et al.*^115^ used ^17^O NMR to study Ti_8_O_8_(OOCR)_16_ titanium oxo clusters, potential building blocks for Ti-based MOFs, with NMR resolving signals from the μ_3_-oxo, μ_2_-oxo and μ_2_-carboxylate oxygen atoms. Liquid-state ^17^O NMR spectroscopy has also been used (in combination with ^31^P and ^183^W NMR) to follow the synthesis of the Cu-based MOF HKUST-1 in the presence of a Keggin-type heteropolyacid (H_3_PW_12_O_40_).^116^ NMR showed that the Keggin anion acts as a template within the solution, first allowing Cu^2+^ ions to attach to this building unit, before the introduction of the trimesic acid linker initiates the formation of the paddle-wheel framework. Addition of Cu^2+^ to PW_12_O_40_^3−^ had a significant effect on the ^17^O NMR spectrum, resulting in several new resonances and confirming quantitative binding of each Cu^2+^ to one anion. The ratio of the intensities of the ^17^O resonances suggest the Cu^2+^ ions bind preferentially to terminal oxygen atoms within the Keggin ion. The greater chemical flexibility of MOF provides opportunities to tailor the pore size and shape to a much greater extent and, when coupled with the presence of open metal coordination sites, leads to a wide range of applications in adsorption and storage. In addition to enriching the framework, information can also be obtained from ^17^O-enriched guests. For example, Wang *et al.*^117^ investigated the dynamics of C^17^O_2_ within Mg- and Zn-CPO-27 using variable temperature ^17^O NMR of static samples. Changes in the ^17^O NMR spectrum of C^17^O_2_ loaded into the framework were seen owing to reduced dynamics as the temperature decreased. These changes were compared to those in lineshapes simulated^118^ assuming different types and rates of motion, and revealed that they results from changes in the ‘wobbling’ of CO_2_ adsorbed to the metal centre and non-localised ‘hopping’ of molecules between different metal sites, and were able to provide parameters to define the two types of motion. The changes with temperature of the CO_2_ wobbling and hopping angles were more pronounced for Zn-CPO-27 than the Mg analogue, confirming the relatively weaker Zn–O binding. Three different enrichment strategies were used in recent work on loading of nanoparticles (Cu and ZnO) within MOF-5;^106^ including selective enrichment of the terephthalate linker prior to synthesis, of the Zn_4_O metal cluster (*via* use of H_2_^17^O(l) in the synthesis) and simultaneous enrichment of both. No isotopic exchange between the two components was observed during MOF formation. ^17^O was also incorporated into the ZnO nanoparticles *via* the *in situ* reaction of ZnEt_2_ with H_2_^17^O(l) inside the MOF pores. ^17^O NMR was then used to verify the presence of the nanoparticle inside the pore but no exchange between the nanoparticle and the MOF-5 framework was seen, suggesting relatively little interaction between the two.

## Outlook

The goal for developing advanced structural characterisation techniques such as NMR spectroscopy is to better understand both the structure of materials and their reactivity. In the case of the porous materials discussed in this Perspective, the fact that oxygen atoms are important in the connectivity of structures of almost all zeolites and MOFs means ^17^O NMR is ideal for studying the local structure. This is particularly important in cases of structural disorder where information from NMR spectra can be vital because other techniques, such as X-ray diffraction, do not provide the same atomic-scale insight. NMR spectroscopy can also give important information in cases where changes in the local structure leads to flexibility in the material as a whole.

However, there are significant remaining challenges. While there is no doubt that NMR spectra contain a wealth of structural information, they are sometimes difficult to obtain experimentally and often complex to interpret. For ^17^O NMR, the challenges of its low natural abundance and low sensitivity have restricted its widespread and routine use for the study of porous solids, despite its considerable promise. We have shown in this Perspective that there have been recent advances in the design of new and more efficient methods for enrichment of microporous materials, but that understanding in detail how this enrichment works (and the implications for the isotope distribution between and within products) is by no means a generally solved problem. Whilst undoubtedly a potential challenge and complication, the potential selectivity of isotopic enrichment also offers a huge opportunity to understand reactivity and mechanism.

It might also be necessary to ask whether isotopic enrichment and the modifications to synthetic procedures (and possible even also to the final products) that this requires is actually worth the cost and effort. Progress in dynamic nuclear polarization (DNP) technology over the last few decades has advanced this technique to the point where many believe it is the best (or even the only viable) option for the future development of NMR spectroscopy.^119^ The transfer of magnetisation from electrons to nuclei of interest in the system has the potential to yield vast signal enhancements (up to a theoretical value of 660 for ^1^H, with potential savings of factors of over 430 000 in experimental time). Not only could DNP give spectra with much greater sensitivity, enhancements of a magnitude similar to the theoretical maximum would make it possible to run experiments that simply aren't possible at natural abundance. A number of ^17^O DNP experiments have been carried out on non-porous solids (often also on enriched samples),^119^ but the first example of natural abundance ^17^O DNP experiment on a MOF (Zr-containing MIP-206) has recently been shown.^120^ A DNP enhancement of 28 was seen for ^1^H, with magnetisation then transferred to ^17^O using CP, enabling a MAS NMR spectrum of 30 mg of sample to be obtained in 48 hours. While not possible at natural abundance without DNP, this does highlight that there is still a long way to go – much greater enhancements are needed to perform the multi-dimensional and multi-nuclear experiments that will be needed to understand the detailed structure of porous solids. It is probably also worth noting that even an ^17^O enrichment level of ∼4% (reasonably routine in many approaches) represents an increase in sensitivity of a factor of 100, and substantial savings in time. Whilst enrichment is reasonably costly, the advanced equipment required for DNP comes at a very high capital and running costs at present. It will be interesting to see how ^17^O DNP develops and improves over the next few years – if direct (*i.e.*, transfer to ^17^O) or indirect (*i.e.*, *via* CP from ^1^H) DNP becomes more popular or practical and how applicable DNP is to materials with different pore sizes and shapes (and whether particularly small pore volumes limit the sensitivity gain). While post-synthetic isotopic enrichment can potentially change the structure or order in a material, and adaptions to enable enrichment *in situ* during synthesis can potentially change the final product formed, for microporous materials in particular the need to introduce a radical solution in DNP can have a significant impact on the pore structure and host–guest interactions, in some cases limiting the specific forms of porous solids that can be studied. There is much to gain with DNP in the study of microporous materials, but also much left to investigate and control. DNP is not without its complications – the complex spin dynamics of CP transfer to ^17^O (particularly when relaxation is rapid as for many OH groups), the potential surface selectivity and the lack of quantitative enhancement make accurate and quantitative measurements a more difficult task. It seems likely that in the medium term at least there is a place for isotopic enrichment and conventional NMR experiments as well as increased DNP investigations. In fact, it may well be that the most fruitful avenue is combination of the two – maximising sensitivity and enabling the selectivity inherent in both approaches to provide more information on structure and reactivity.

The challenges of interpreting and assigning signals in experimental NMR spectra of solids is considerable but has been eased in recent years by the concomitant use of computation. As the level and types of disorder present increases, the problem becomes how a sufficiently large, chemically sensible and relevant set of structural models can be generated efficiently, ensuring time is not wasted computing parameters for structures related by symmetry. As described above, progress has been made by using automated algorithms to generate all possible atomic arrangements or crystal structure prediction and structure searching. The very high sensitivity of NMR to very small changes in the local structure requires high accuracy both in structural optimisation and the calculation of NMR parameters. However, the high cost of first-principles calculations restricts the number of structural models it is feasible to study and limits study as the size of the system (and the level and type(s) of disorder) increases. For significant progress to be made future research must be focussed on the development of methods to restrict the chemical, structural and energy space that needs to be considered, and/or on carrying out such searches in a more efficient manner. It will be interesting to see how, and if, automation and machine learning can play a role here. Recent work on chemical shifts in molecular organic systems has shown that machine learning approaches can predict this parameter to within DFT accuracy by considering just the local environment.^121^ If this could be translated into inorganic systems, and were applicable to a much wider range of nuclei, this would allow very rapid analysis of huge suites of structures, enabling the most relevant to be selected for further study. While clearly a huge challenge, it is possible that a combination of NMR, machine learning and DFT could be used in the future in an automated structural refinement process akin to that routinely implemented in powder XRD. It is likely that such an ambitious aim could ultimately be limited by the uncertainty associated with optimised geometries and predicted NMR parameters as a result of the inherent inaccuracy of the functionals used in DFT. Any step change in this accuracy (whilst retaining transferability) could transform the use of computation in NMR and, ultimately, the role NMR can play in the structural characterisation of porous solids. The progress made to date and the growing use of NMR crystallographic approaches suggests this is an area worth pursuing, and one where the reward is potentially great.

Perhaps even more important in the longer term is the potential for ^17^O NMR spectroscopy to be used to follow the reactivity of porous materials, giving us an enhanced understanding of how they work in practice. The fact that oxygen atoms line the internal surfaces of many porous solids, and in particular zeolites, leads to ^17^O NMR being the ideal probe to follow local environment changes caused by, for example, interactions with guest species inside the pores. The technical challenges of rapid MAS (*i.e.*, small rotor volumes, the need for very stable spinning and the restricted temperature range that can be accessed routinely) limit the conditions under which *in situ* and *in operando* studies can be carried out and, therefore, limit the ultimate relevance to real–life processes. For ^17^O, there is the added complication of quadrupolar broadening and the need for more complex (and longer) experiments if high-resolution spectra are required to extract site-specific structural information. Easier approaches include experiments on static samples, where there is significantly more flexibility to add reagents and to vary temperature and pressure, or the use of *ex situ* sampling of reactions. The former could suffer from a lack of resolution, depending on the reaction and species studied, while the latter has the danger that changes to the sample are likely to occur during its preparation and transfer. A recent review by Jaegers *et al.*^122^ describes recent progress in *in operando* MAS NMR of catalysts, the introduction of new rotor designs that can handle high temperatures and high pressures in fast MAS experiments with a check valve for direct gas loading, and the development of new continuous flow MAS probes. While these technologies are at an early stage, initial results are promising and it is hoped that these approaches will not only be applicable to ^17^O NMR in the future but will be able to exploit the additional information that use of selectively enriched materials or reagents can bring.

We are at an interesting and exciting stage in ^17^O solid-state NMR spectroscopy of microporous materials – this Perspective shows significant progress has been made in the development of new chemistry for enrichment, in NMR methodology and in the interpretation and analysis of complex spectral lineshapes. Research over the last few years has given us tantalising glimpses into the level of detailed structural information that can be obtained. The field is now in a position to fully exploit the transformative developments in new hardware, advanced computing and new polarisation technologies that will inevitably be seen over the next decade, resulting in the question of when, rather than if, ^17^O NMR will become a vital but routine step in the characterisation of porous solids.

## Conflicts of interest

There are no conflicts to declare.
